# Semi-automated assessment of the risk of bias due to missing evidence in network meta-analysis: a guidance paper for the ROB-MEN web-application

**DOI:** 10.1186/s12874-023-02038-9

**Published:** 2023-10-07

**Authors:** Virginia Chiocchia, Alexander Holloway, Georgia Salanti

**Affiliations:** Institute of Social and Preventive Medicine, University of Bern, Bern, Switzerland

## Abstract

Network meta-analysis compares multiple interventions and estimates the relative treatment effects between all interventions, combining both direct and indirect evidence. Recently, a framework was developed to assess the Risk Of Bias due to Missing Evidence in Network meta-analysis (ROB-MEN) which is part of the more comprehensive framework to evaluate the Confidence In the evidence for Network Meta-Analysis (CINeMA). To produce an overall risk of bias judgement for each network estimate, ROB-MEN: performs an assessment of the bias due to missing evidence in each possible pairwise comparison; combines the assessment with the contribution from the direct pairwise comparisons; considers the potential for small-study effects. To facilitate and semi-automate this process, ROB-MEN has been implemented in a user-friendly web-application (https://cinema.ispm.unibe.ch/rob-men). Here we provide a tutorial detailing the functionality and use of the application consisting of data upload, analysis configuration, output visualisation, and production of the tool’s output tables for recording the risk of bias assessment. We also illustrate an example application using the demo dataset available for download on the application’s homepage. The ROB-MEN web-application is open-source and freely available (https://github.com/esm-ispm-unibe-ch/rob-men).

**Supplementary Information:**

The online version contains supplementary material available at 10.1186/s12874-023-02038-9.

## Background

Results obtained from evidence synthesis methods can be compromised by the presence of selective outcome reporting or publication bias [1]. Such bias due to missing evidence also applies to network meta-analysis (NMA) which compares multiple interventions by combining both direct and indirect evidence. A rigorous methodology to evaluate this bias was lacking until recently, when the ROB-MEN (risk of bias due to missing evidence in network meta-analysis) framework was developed to address this issue [2]. ROB-MEN considers first the bias due to missing evidence in each possible pairwise comparison in the network by performing a within-study and an across-study bias assessment. Based on these two assessments, a level of *No bias detected* or *Suspected bias favouring X (intervention)* is assigned to each comparison. This is then combined with the contribution from the direct comparisons [3] and the potential for small-study effects to obtain an overall risk of bias judgement (*High risk*, *Some concerns*, or *Low risk*) for each network estimate. ROB-MEN is part of the more comprehensive framework to evaluate the confidence in the evidence for network meta-analysis results (CINeMA) [4, 5].

The complex and larger structure of a network of studies makes the bias assessment process longer and more labour-intensive than for a pairwise meta-analysis. For this reason, some of the ROB-MEN method’s steps have been semi-automated in a user-friendly web-application available at https://cinema.ispm.unibe.ch/rob-men/. While the methodology underpinning ROB-MEN and the algorithm to automate the final bias judgements are detailed in the original publication [2], here we describe practical usage guidelines and technical details about the application itself—the expected data format, the available analysis options, and the decision rules implemented in the software. We also present an illustrative example using the demo dataset available on the ROB-MEN website, relative to an NMA of non-invasive diagnostic tests for the detection of coronary artery disease [6].

## Implementation

The ROB-MEN method is implemented as a multi-page web-application with a workflow that intuitively guides users through the process of analysing their data. The web-application is implemented using R Shiny (7), Javascript, and Bootstrap. Once users upload their data, all analysis is performed server-side, and so the only pre-requisite for use of the application is access to a modern web browser. Users are also able to download the application’s source files (https://github.com/esm-ispm-unibe-ch/rob-men) and run a local instance of the web-application, but this requires additional environment configuration steps and thus is only recommended for advanced users.

ROB-MEN runs the R packages *netmeta* [8] and *BUGSnet* [9] in the background to perform frequentist and Bayesian NMA, respectively. User-written R functions to run Bayesian network meta-analysis and meta-regression are used when the meta-analysis synthesises standardised mean differences (SMD). These functions are based on previous user-written functions (available at https://github.com/esm-ispm-unibe-ch/NMAJags) and uses the same model parameterisation described in the *crossnma* package [10, 11].

## User interface

### Data upload

Data is uploaded in the ‘Load’ page as a plaintext, delimited file. The dataset must be in arm-based format, where each row corresponds to a single study arm. The dataset can also include variables (data columns) not required by the app but those needed must be named and formatted as reported in the instructions available on the app homepage. The required data columns are:

- *id*: a unique identifier for the studies;
- *study*: the name of the studies, also uniquely identifiable;
- *t*: the treatment code (or name) in each arm of the studies;
- *n*: the sample size in each arm of the studies; this must be reported for all studies including those with missing outcome values;
- The outcome data column(s): *mean* and *sd*, or *r*, for continuous and dichotomous outcomes, respectively. These report, in order, the mean and standard deviation of the continuous outcome, or the number of events of the dichotomous outcome (numbers experiencing the outcome). The dataset can have these data columns for only one outcome of interest.

The dataset should include all studies identified in the systematic review regardless of whether they report the outcome of interest or not, in order to evaluate selective non-reporting of the outcome being investigated in the current network meta-analysis. If a study does not report the outcome of interest, the outcome data columns should have missing values, reported as non-numeric values such as asterisks (*), NA or simply left blank.

Once the dataset file is uploaded, the data is displayed in the ‘Load’ page. Users should ensure the format of their data matches that of the examples provided. An example of the arm-based format for the dataset to upload is shown in Table 1.

**Table 1:** Data format for upload from the NMA example of non-invasive diagnostic tests

id	study	t	n	r
26,764,061	R1. Dedic A	CCTA	250	41
26,764,061	R1. Dedic A	Standard care	250	31
26,052,677	R2. Levsky JM	CCTA	200	30
26,052,677	R2. Levsky JM	SPECT-MPI	200	32
25,466,568	R3. Hamilton-Craig C	CCTA	322	26
25,466,568	R3. Hamilton-Craig C	Exercise ECG	240	9
23,998,546	R4, R5. Linde JJ	CCTA	299	49
23,998,546	R4, R5. Linde JJ	Standard care	301	36
24,026,478	R6. Lim SH	SPECT-MPI	1126	73
24,026,478	R6. Lim SH	Standard care	564	56
23,664,718	R7. Miller CD	CMR	52	5
23,664,718	R7. Miller CD	Standard care	53	11
22,830,462	R8. Hoffmann U	CCTA	501	59
22,830,462	R8. Hoffmann U	Standard care	499	40

Data reported only for the first 8 studies. id and study specify the study; t specifies the treatment; n indicates the sample size; r indicates the number of events. CCTA: coronary computed tomographic angiography; SPECT-MPI: single-photon emission computed tomography-myocardial perfusion imaging; ECG: electrocardiogram; CMR: cardiovascular magnetic resonance

### Analysis

Once the data is uploaded, the user can proceed to the ‘Analyse’ page to configure the parameters for the analyses. The user must choose the effect size pertinent to the type of outcome data; odds ratios (OR) or risk ratios (RR) for dichotomous outcomes, and mean difference (MD) or standardised mean differences (SMD) for continuous outcomes. The user must also specify whether smaller outcome values are *desirable* (e.g. for outcomes such as mortality) or *undesirable* (e.g. scales or scores where higher values indicate improvement).

Next, the user selects the synthesis model (*random* or *common effects*) and the reference treatment. The Bayesian analyses are performed using two Markov chains, while the priors on the baseline treatment effects, relative treatment effects, their variance, and the meta-regression interaction coefficients are those defined as default in the *BUGSnet* package [9].

The final parameters to select refer to the Bayesian network meta-regression (NMR) analysis that uses the study variance as a covariate. By default, the *thinning factor* is set to 1, and the *burn-ins* and *iterations* for the Markov chain Monte Carlo (MCMC) simulations are 1,000 and 10,000, respectively, with the burn-in number automatically set to be at least 10% of the number of iterations.

The user has the option to choose between *unrelated* and *exchangeable* treatment-specific interaction coefficients. We recommend choosing the less strict assumption of *unrelated* coefficients, meaning that the “basic” interaction terms (${\beta }_{i}$ regression coefficients of each treatment $i$versus the reference treatment) are independent of each other and are given unrelated vague priors [12]. However, this option can be computationally intensive for very large networks due to the large number of parameters to estimate; for T treatments we need to estimate T-1 basic parameters for the treatment effects and T-1 basic parameters for the treatment-specific covariate effects (plus heterogeneity parameters). If the data is not enough to estimate so many parameters, the synthesis model might not converge, and estimation might fail altogether. In these cases, the alternative assumption of *exchangeable* interaction terms can be chosen, where the regression coefficients ${\beta }_{i}$ are “related” in the sense that they are drawn from a normal distribution with common mean and between-treatment variance, ${\beta }_{i} \sim N(b, {\tau }^{2})$. Note that this assumption only makes sense for networks with a natural reference treatment (such as placebo), as specified by the reference treatment parameter above.

The NMR model specification parameters can be changed in case of non-convergence of the MCMC which can be detected in the upper part of the ‘Bayesian network-meta regression’ tab after the analysis is completed. To change any of the analysis options after the analysis has started or the output is produced, the app must be re-loaded, and the data uploaded again.

Once all the options have been selected the app will run the required analyses in the background as the user presses the *Start Analysis* button. The process can take up to a few minutes depending on the size of the network and the amount of data.

#### Output of analyses

Once the analyses are completed the app will display different outputs in separate tabs under the main ‘Analyse’ page. Characteristics of the network, interventions and direct comparisons are summarised in the ‘Data summary’ tab. The outputs from NMA, including forest plot and league table, are presented under the ‘Bayesian network meta-analysis’ tab. Similarly, the results from the NMR are reported in the ‘Bayesian network meta-regression’ tab. The NMA and NMR outputs are reported merely for reference for the user. The unadjusted and adjusted relative treatment effects, from NMA and NMR respectively, are automatically reported in the ROB-MEN for the assessment of small-study effects at the network level.

In the ‘Bayesian network meta-regression’ tab, the user can check the MCMC for convergence of the NMR model by looking at the values of the Rubin-Gelman statistic ($\widehat{R}$), with values close to 1 indicating convergence, or by downloading and inspecting the trace plots, where convergence is indicated by chains well distributed around a mean value [13]. As explained in the previous section, in case of non-convergence, the user should re-run the analysis with an increased number of burn-ins and iterations or, in case of high autocorrelation, try a different thinning factor. If relevant for very large networks, changing the interaction terms assumption to *exchangeable* may also help. For more details on MCMC convergence and/or performing Bayesian analysis the user should refer to the appropriate literature [13] or seek expert advice. This tab also gives the values of the “basic” regression coefficients (Betas, ${\beta }_{i}$) for the interaction between relative treatment effects and study variance [12]. If *unrelated* treatment-specific interactions were chosen, it will display as many Betas as “basic” treatment-specific interactions. Otherwise, for the *exchangeable* option, it will display the mean $b$ of the Normal distribution from which the coefficients are drawn. The network meta-regression plots can also be downloaded as PDF files: each line shows how the linear effect of each treatment versus the reference changes for different study variances. Finally, the league table displays the relative treatment effects at the covariate value of minimum observed variance.

If there are comparisons with at least 10 studies, the tab ‘Funnel plots and test for small-study effects’ will show contour-enhanced funnel plots and test for small-study effects for such comparisons [14–17], which can aid the across-study assessment of bias in the Pairwise Comparisons Table. In cases where many funnel plots are available, only the first six are shown. For better visualisation, the user can download all of them in a PDF by pressing the relevant button.

The contribution matrix [3], key to the assessment of bias in the network estimates, is reported in the tab with the same name. The cells show the contribution that each direct comparison (in the column) make to each network estimate (in the row). This output is also reported purely for reference for the user, since the contribution to the network estimate is combined automatically with the overall judgements that will be made for the pairwise comparisons.

### Pairwise comparison table

Once the analysis is completed, the user can move to the ‘Pairwise Comparisons Table’ page to assess the risk of bias due to missing evidence in each of the possible pairwise comparisons that can be made between the interventions in the network. These are displayed as rows of the Pairwise Comparisons Table and automatically grouped them as follows:

- Group A (observed for this outcome): comparisons with studies reporting the outcome of interest i.e. the direct evidence in the network, and edges in the network graph in the ‘Data Summary’ tab.
- Group B (observed for other outcomes): comparisons with studies only reporting other outcomes, but not the outcome of interest.
- Group C (unobserved): comparisons not identified in the literature i.e. no studies investigating such comparisons were found.

Under the “Number of studies in each comparison” heading, the app calculates and reports automatically, for each comparison, the total number of studies with results for the current outcome or any outcome. These columns also report, in brackets, the total sample size by adding up all participants randomised in the studies investigating the specific comparison for that outcome. By definition, the unobserved comparisons (Group C) have zero in both columns, and those observed for other outcomes (Group B) have zero under the “Reporting this outcome” heading.

#### Within-study assessment of bias

The information reported in the columns described above is useful for the assessment of bias due to selective non-reporting of results within studies, also commonly known as *selective outcome reporting bias*, as it concerns studies identified in the systematic review but missing from the synthesis i.e. the studies report only on other outcomes and not the outcome of interest. The user should first assess the presence of selective non-reporting of results in each study, then, for each observed comparison (Groups A and B), the impact of the missing results across all studies is evaluated to reach a judgement of *no bias detected* or *suspected bias favouring X*. This is facilitated by the signalling questions described in the ROB-MEN framework [2].

The final judgement must be selected, with the relevant direction of bias, for each comparison in the column “Within-study assessment of bias”. This action may be facilitated by setting all of them to “No bias” by pressing the button under the relevant heading and then, manually change those for which there is suspected bias favouring one intervention over the other.

#### Across-study assessment of bias

Unlike the previous assessment, the across-study assessment of bias, commonly known as *publication bias*, applies to all pairwise comparisons, including the unobserved ones (Group C). This is done primarily considering qualitative conditions as described in the ROB-MEN paper [2] and in the Cochrane Handbook [1]. Additionally, for any comparison with at least 10 studies, the user can also consider quantitative techniques for pairwise meta-analysis [14–21]. If applicable, contour-enhanced funnel plots and relevant test for small-study effects are produced in the ROB-MEN app and shown in ‘Funnel plots and test for small-study effects’ tab under the ‘Analyse’ page. Due to space limitations in the webpage the tab only shows up to six plots but, if there are more than six comparisons with 10 or more studies, all funnel plots are displayed in the downloadable PDF file.

Like the previous assessment, the action of selecting the final judgement of *no bias detected* or *suspected bias favouring X* for each comparison in the column “Across-study assessment of bias” may be facilitated by selecting the button *set all to “No bias”* and then, manually adjusting the levels, where needed.

#### Overall judgement

By selecting the *calculate overall judgement* button the overall risk of bias is assigned automatically to each comparison, reflecting the risk of bias levels from the within-study and the across-study assessment of bias. Specifically, if *suspected bias favouring X* was selected in either of the two assessments, this will also be the overall judgement for that comparison. For unobserved comparisons (group C), this will be the level selected in the across-study assessment of bias.

The whole Pairwise Comparison table can be exported as a .csv file by pressing the download button on the top-left corner of the page.

### ROB-MEN table

The next step for the user is the evaluation of the risk of bias due to missing evidence in the network estimate, which represents the main output of interest of the ROB-MEN tool. This assessment is recorded in the ROB-MEN Table which can be accessed in the page with the same name. Here the network estimates are automatically organised into two groups depending on whether the relative treatment effect for that contrast is estimated using direct and/or indirect evidence. The groups are called “mixed/only direct” and “only indirect”, respectively.

#### Evaluation of contribution from evidence with suspected bias

The first assessment to record in the ROB-MEN Table is about the contribution of comparisons with suspected bias to the estimates. By combining the overall judgements assigned to the direct comparisons (Group A) in the Pairwise Comparisons Table and the contribution matrix (reported in the ‘Analyse’ page), the app calculates the total percentage contribution coming from direct comparisons at suspected bias in either direction (i.e. favouring the first or second treatment of the contrast) and automatically report them in the first two columns of the ROB-MEN Table. Based on these values, the user reports their evaluation judgement in the third column “Evaluation of contribution from evidence with suspected bias” by choosing among four possible levels from the drop-down menu:

- *No substantial contribution from bias*: there is no substantial contribution from evidence with bias favouring one of the two treatments;
- *Substantial contribution from bias balanced*: there is a substantial contribution from evidence with suspected bias, but the bias contributions favouring one or the other treatment are somewhat similar;
- *Substantial contribution from bias favouring one treatment*: there is a substantial contribution from evidence with bias favouring one of the two treatments.

What percentage of contribution constitutes a “substantial” amount and when the contributions favouring one or the other treatment are considered “similar” is subjective and decided by the user, but it should be consistent (i.e. the same amount) for all network estimates.

A *set all to “No substantial contribution”* button is also provided for convenience.

#### Bias assessment for indirect evidence

Indirect estimates are calculated from sources of direct evidence with a specific contribution to each contrast as shown in the contribution matrix. The absence of direct evidence for these indirect comparisons may lead to bias if any studies are missing for reasons associated with their results, so this additional source of bias needs to be considered for indirect estimates. This is represented by the overall judgement of risk of bias for pairwise comparisons *observed for other outcomes* or *unobserved* (Group B and C) in the Pairwise Comparisons Table, which is automatically copied in the relevant column of the ROB-MEN Table. Therefore, no action is needed from the user in this step.

Note that the copied judgement is greyed out for the “mixed/only direct” estimates as this specific bias assessment only applies to the indirect estimates.

#### Evaluation of small-study effects

The columns “NMA treatment effect” and “NMR treatment effect at the smallest observed variance” report the point estimates and 95% credible intervals also provided in the league tables of the respective Bayesian analyses in the ‘Analyse’ page. These are used to evaluate any small-study effects, by looking at the difference of the two estimates and the overlap of their credible intervals, as described in the original ROB-MEN publication [2].

The user reports their judgement in the penultimate column of the ROB-MEN Table, by choosing between three levels from the drop-down menu:

- *No evidence of small-study effects*: there is no indication of small-study effects for the estimate;
- *Evidence of small-study effects favouring one treatment*: there is an indication that one of the two treatments is favoured by the small studies.

Again, for a faster selection, the user can press the *set all to “No evidence”* button and then manually change it for those estimates where there is evidence of small-study effects, if any.

#### Overall risk of bias and connection with CINeMA software

The user can assign the overall risk of bias due to missing evidence to each network estimate by pressing the *calculate overall RoB* button under the heading of the last column, where this is reported. The overall risk of bias judgement is calculated according to the ROB-MEN algorithm, described in Table 2, and can take a level of *Low risk*, *Some concerns* or *High risk*, as in the CINeMA framework domains. The app allows the user to manually change the level if they do not want to follow the proposed rules and use instead “stricter” or “more relaxed” approaches. If the rules are overridden, we recommend justifying and clearly describing the rationale for the manual changes.

The ROB-MEN Table can be exported as a .csv file using the download button in the top-left corner of the page. The downloaded table is formatted such that it can be uploaded in the Reporting Bias domain of the CINeMA web-application (https://cinema.ispm.unibe.ch/), which will automatically show the risk of bias levels for each network estimate in the standard CINeMA output.

**Table 2:** ROB-MEN algorithm to calculate overall risk of bias for the network estimates

**Low risk**	There is no substantial contribution from evidence with suspected bias favouring one of the two treatments, OR There is substantial contribution from evidence at suspected bias, but it is split more or less equally between evidence with bias favouring one of the treatments and evidence with bias favouring the other treatment **AND** There is no evidence of small-study effects favouring one of the two treatments, OR [*For indirect estimates only*] There is no suspected bias favouring one of the two treatments from the assessment of indirect evidence.
**Some concerns**	All other combinations
**High risk**	There is substantial contribution from evidence with suspected bias favouring one of the two treatments **AND** There is evidence of small-study effects favouring the same treatment, OR [*For indirect estimates only*] There is suspected bias favouring that treatment X from the assessment of indirect evidence.

## Case study

We report in this section how to implement in the app the ROB-MEN assessment for the NMA of randomised controlled trials comparing non-invasive diagnostic strategies for the detection of coronary artery disease in patients with low risk acute coronary syndrome (6). The formal assessment process is described in the original ROB-MEN tool publication (2). Interested readers are able to follow along with the same dataset used here, which can be downloaded directly from the ROB-MEN application’s homepage (https://cinema.ispm.unibe.ch/rob-men/).

First, we upload the .csv file in the ‘Load’ page, which also displays the dataset. The network for the outcome of interest, referral to coronary angiography, included 18 studies comparing 6 different interventions: exercise electrocardiogram (ECG), single-photon emission computed tomography-myocardial perfusion imaging (SPECT-MPI), coronary computed tomographic angiography (CCTA), cardiovascular magnetic resonance (CMR), stress echocardiography (Stress echo), and standard care.

Part of this demo dataset is shown in Table 1 as an example of arm-based format dataset to upload.

**Fig. 1:**
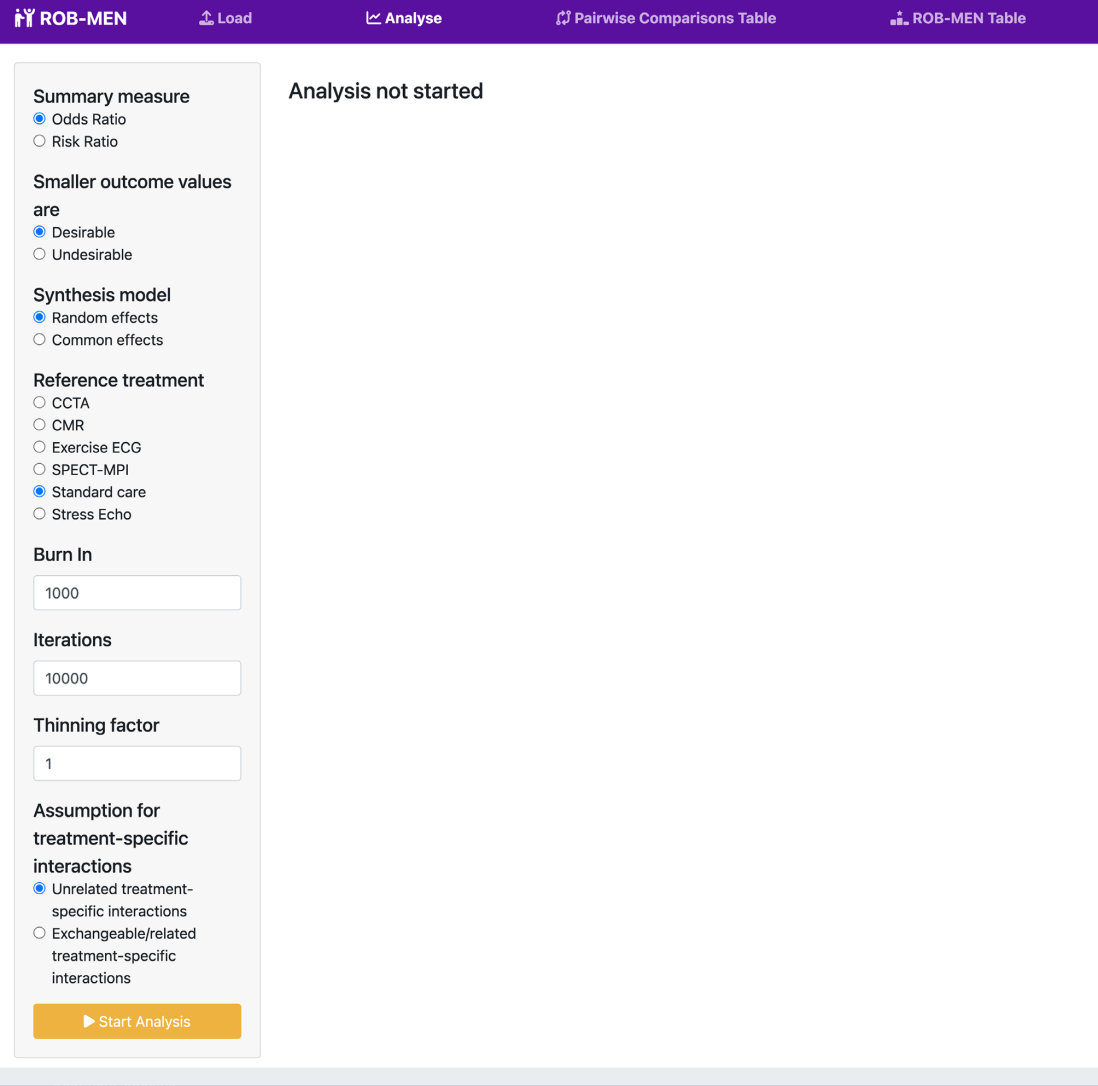
Screenshot displaying the analysis configuration page for the NMA example of non-invasive diagnostic tests

In the ‘Analyse’ page, we select *odds ratio* as summary measure, *random effects* as synthesis model and *standard care* as reference treatment. Since the outcome is referral to invasive coronary angiography, smaller outcome values are *desirable*. For the NMR model we use the default values for the burn-in, number of iterations and thinning factor, and we select *unrelated treatment-specific interactions* (Fig. 1), then begin the analysis.

**Fig. 2:**
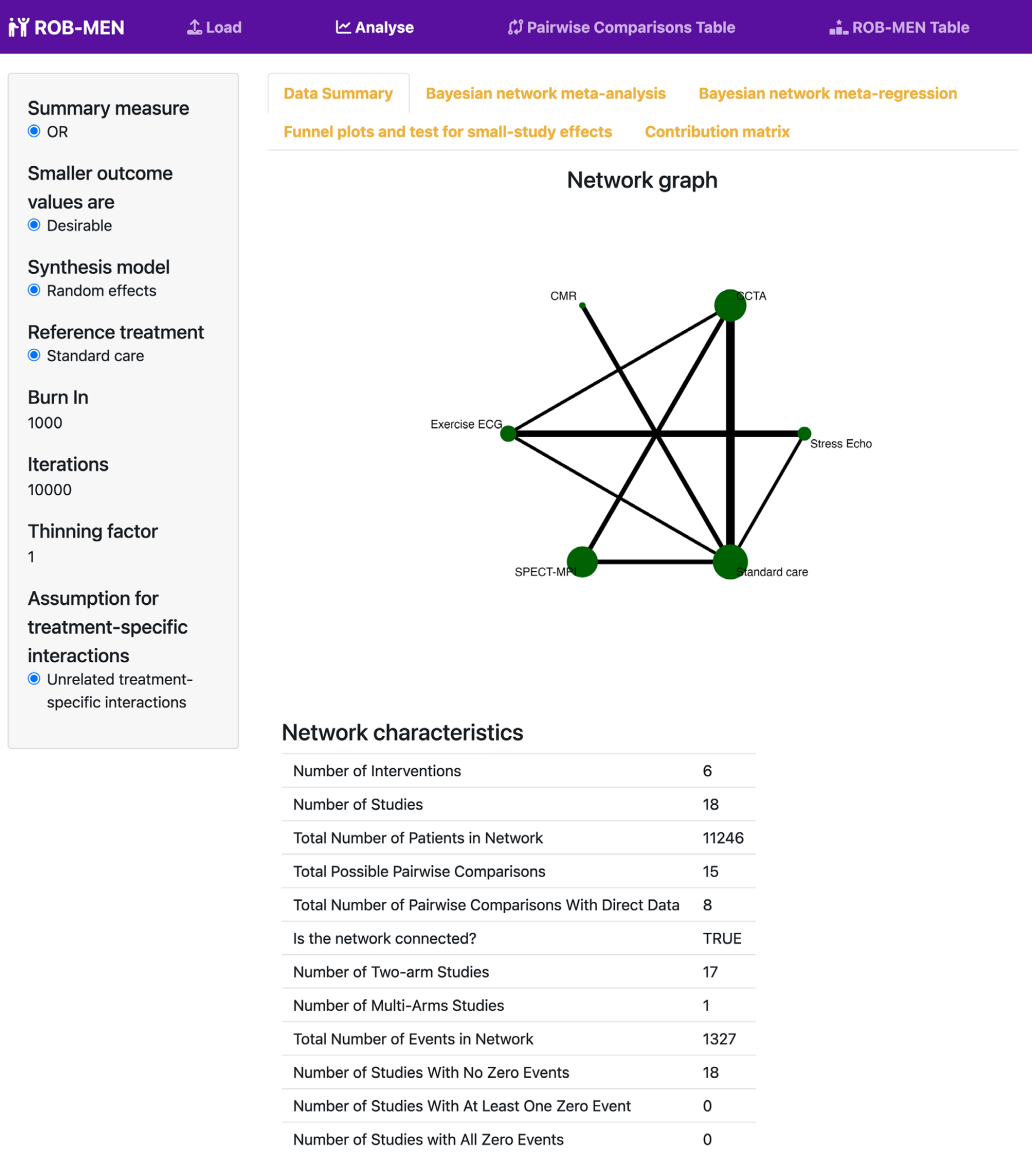
Screenshot displaying the Data Summary page for the NMA example of non-invasive diagnostic tests

Once the analysis is completed, the ‘Analyse’ page displays a summary of the network, intervention, and direct comparison characteristics (Fig. 2), as well as the results of the analyses (Additional files 1 and 2). We can verify that the NMR model converged in the ‘Bayesian network meta-regression’ tab, as the values of the Gelman-Rubin statistic are all very close to 1 (Additional file 2) and the trace plots look well distributed (not shown). In this instance, funnel plots and test for small-study effects are not available because all comparisons have less than 10 studies. The contribution matrix is also displayed.

**Fig. 3:**
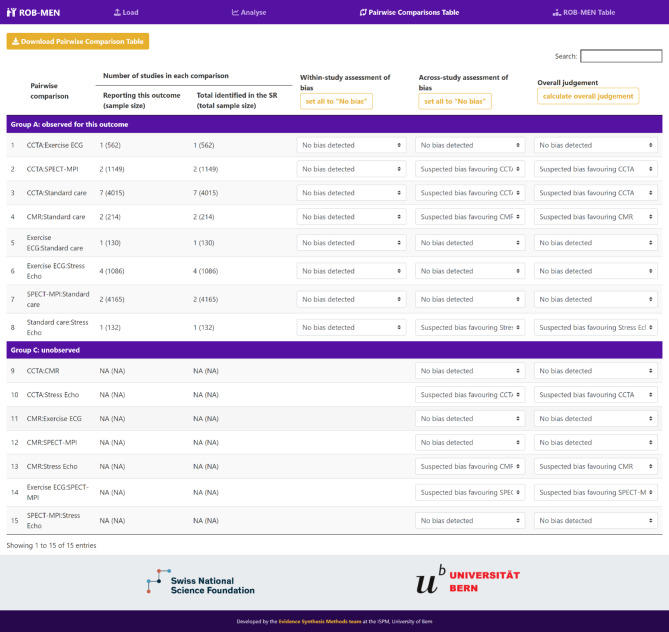
Screenshot displaying the Pairwise Comparison Table for the NMA example of non-invasive diagnostic tests

In the Pairwise Comparison Table, the 15 possible pairwise comparisons are grouped according to the data availability: 8 were observed for the outcome of interest, referral to coronary angiography, (Group A), while the remaining 7 were unobserved (Group C), i.e. no comparisons were observed only for other outcomes (Fig. 3). Since in this network there are no additional studies that did not report results for referral to coronary angiography, we can click on the *set all to “No bias”* button under the “Within-study assessment of bias” heading to automatically assign “No bias detected” to all observed comparisons. The across-study assessment of bias in this network was informed only by qualitative considerations [2] as none of the comparisons included 10 or more studies so graphical or statistical techniques were not applicable. We first click on the *set all to “No bias”* button under the “Across-study assessment of bias” heading and then manually change the levels for the 7 comparisons judged at *suspected bias* using the drop-down menu in the relevant rows (Fig. 3). The overall risk of bias judgements are automatically assigned to each comparison by clicking the *calculate overall judgement* button under the “Overall judgement” heading. 

**Fig. 4:**
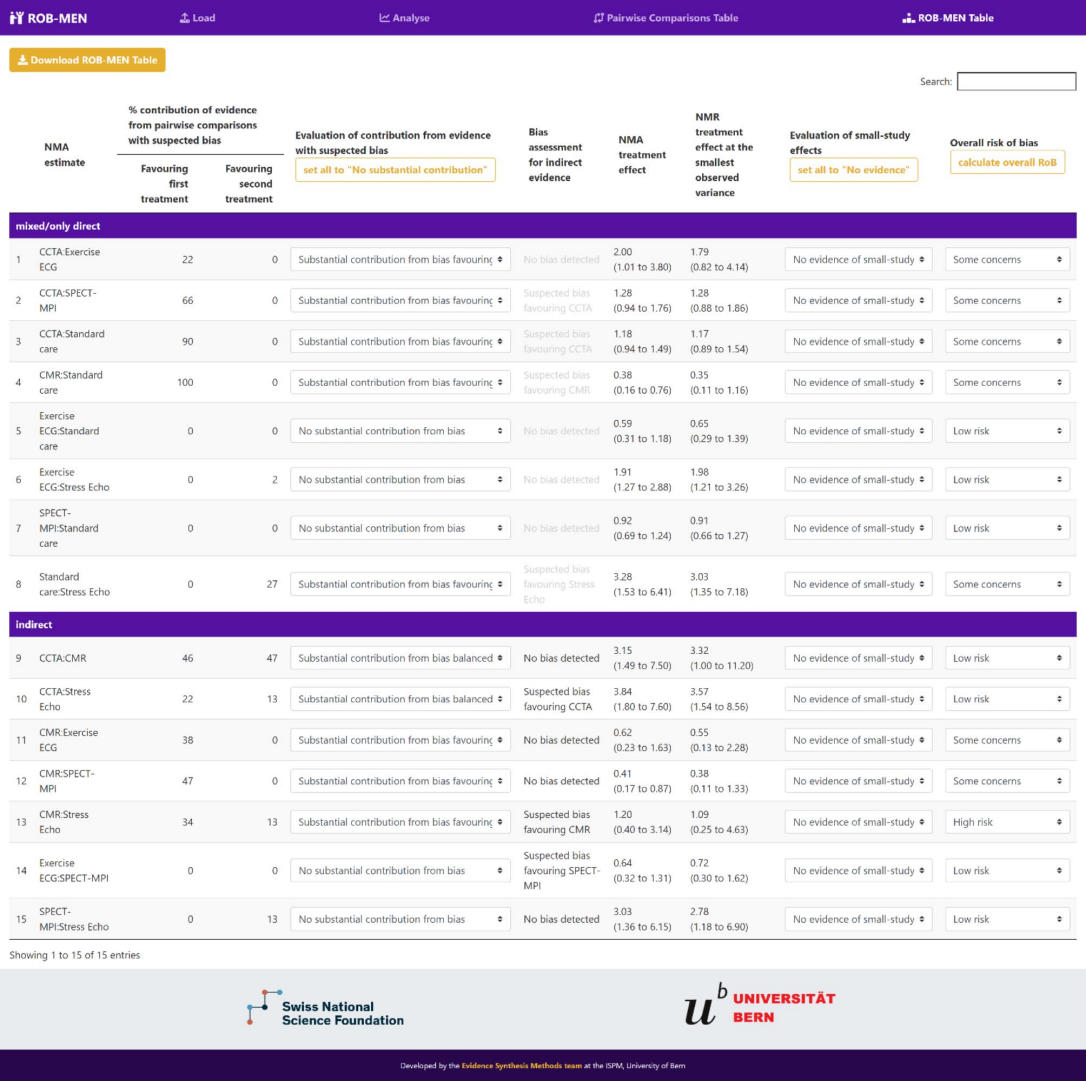
Screenshot displaying the ROB-MEN Table for the NMA example of non-invasive diagnostic tests

The ROB-MEN Table is presented in (Fig. 4). The overall judgements from the last column of the Pairwise Comparisons Table (Fig. 3) have been combined with the percentage contribution of the direct comparisons (available in the ‘Contribution matrix’ tab under ‘Analyse’) to automatically produce the figures in the first two columns of the ROB-MEN table (Fig. 4). For this example, we made the decision that 15% constitutes a substantially biased contribution in favour of one treatment, and we manually select the levels of “*Evaluation of contribution from evidence with suspected bias*” accordingly (third column in ROB-MEN table, Fig. 4). The additional risk of bias for indirect estimates is automatically entered in the “Bias assessment for indirect evidence” column by copying the overall judgements from the last column of the Pairwise Comparisons Table; no input is needed from the user. For the evaluation of small-study effects we compare the estimated relative treatment effects from NMR (see Additional file 2) to those obtained from the original network meta-analysis (see Additional file 1) presented in the “NMA treatment effect” and “NMR treatment effect at the smallest observed variance” columns, respectively. Since all network meta-regression estimates are not very different to the corresponding unadjusted estimates and there is a good overlap between the credible intervals, we apply *No evidence of small-study effects* to all estimates by clicking on the *set all to “No bias”* button under the “Evaluation of small-study effects” heading. Finally, we click the *calculate overall RoB* button under the “Overall risk of bias” heading to assign the overall risk of bias level due to missing evidence which is automatically calculated using the rules set out in Table 2. The completed ROB-MEN Table for the example is shown in the last column of Fig. 4.

## Discussion

We have presented the functionality and use of the ROB-MEN web-application for facilitating the evaluation of the risk of bias due to missing evidence in network meta-analysis. The application semi-automates some of the required steps of the ROB-MEN tool [2], and produces the two output tables in a ready-to-use .csv format. The users should know the underpinning methodological ROB-MEN framework before attempting to use the tool in practice. Indeed, this paper does not substitute the original ROB-MEN publication where the evaluation methods and algorithm are described in detail. To avoid the potential for misuse of the tool, we recommend the following:

- First, familiarise with the theorical aspects of the ROB-MEN tool.
- Only when the ROB-MEN framework is clear, use this paper as a user manual to perform the ROB-MEN assessments in the app.
- Seek expert judgement from a statistician or methodologist when analysis or technical aspects are unclear or not known.

The ROB-MEN application integrates with the existing CINeMA framework (4), with the Reporting Bias domain in the CINeMA being able to make direct use of the application’s output, increasing the speed and ease with which users are able to carry out their entire evaluation.

The aim of the ROB-MEN web-application is to aid the users in the risk of bias assessment procedure. We do not recommend using our app to perform the primary and secondary network meta-analyses from systematic reviews. Appropriate software packages or other specific web-applications [22, 23] should be used for that purpose.

The app, like CINeMA, currently supports only certain types of data, specifically arm-based format for dichotomous and continuous outcomes. Future updates could focus on implementing different data formats and types e.g., time-to-event and count data. Further research would be needed to extend both frameworks to handle other analyses and network types, such as dose-response NMAs, NMAs of diagnostic test accuracy, individual participant data and component NMAs. As for any other process assessing the risk of bias or evaluating the quality of evidence, ROB-MEN also involves subjective decisions. While some degree of subjectivity is inevitable, we tried to limit it by integrating quantitative elements and proposing specific criteria and algorithms. It is, however, unclear how this may affect the agreement between evaluations made different reviewers as the reproducibility of CINeMA and ROB-MEN assessments has not been studied so far. Therefore, future work examining the interrater agreement and the reproducibility of these tools would be informative.

## Conclusion

The ROB-MEN web-application is open-source and freely available and it has already had some improvements from its first released version also thanks to the feedback received by the users. This article provides a step-by-step tutorial to the use of the app and its functionality, and we believe it will prove helpful to the user by speeding up the process and clarifying some aspects. More training material and examples are also linked in the main web-application homepage.

### Electronic supplementary material

Below is the link to the electronic supplementary material.

Supplementary Material 1

Supplementary Material 2

## Data Availability

The dataset analysed during the current study is available to download in the main homepage of the ROB-MEN web-application.

## References

[1] Page MJ, Higgins JP, Sterne JA. Chapter 13: Assessing risk of bias due to missing results in a synthesis. In: Cochrane Handbook for Systematic Reviews of Interventions [Internet]. version 6.0. Cochrane; 2019. Available from: www.training.cochrane.org/handbook.

[2] Chiocchia V, Nikolakopoulou A, Higgins JPT, Page MJ, Papakonstantinou T, Cipriani A (2021). ROB-MEN: a tool to assess risk of bias due to missing evidence in network meta-analysis. BMC Med.

[3] Papakonstantinou T, Nikolakopoulou A, Rücker G, Chaimani A, Schwarzer G, Egger M (2018). Estimating the contribution of studies in network meta-analysis: paths, flows and streams. F1000Res.

[4] Nikolakopoulou A, Higgins JPT, Papakonstantinou T, Chaimani A, Giovane CD, Egger M (2020). CINeMA: an approach for assessing confidence in the results of a network meta-analysis. PLoS Med.

[5] Papakonstantinou T, Nikolakopoulou A, Higgins JPT, Egger M, Salanti G. CINeMA: Software for semiautomated assessment of the confidence in the results of network meta-analysis. Campbell Systematic Reviews [Internet]. 2020 Mar [cited 2021 Aug 17];16(1). Available from: https://onlinelibrary.wiley.com/doi/10.1002/cl2.1080. 10.1002/cl2.1080 PMC8356302 37131978

[6] Siontis GC, Mavridis D, Greenwood JP, Coles B, Nikolakopoulou A, Jüni P et al. Outcomes of non-invasive diagnostic modalities for the detection of coronary artery disease: network meta-analysis of diagnostic randomised controlled trials. BMJ. 2018;k504. 10.1136/bmj.k504 PMC5820645 29467161

[7] Chang W, Cheng J, Allaire JJ, Sievert C, Schloerke B, Xie Y et al. shiny: Web Application Framework for R [Internet]. 2021 [cited 2023 Jul 14]. Available from: https://cran.r-project.org/web/packages/shiny/index.html.

[8] Rücker G, Krahn U, König J, Efthimiou O, Davies A, Papakonstantinou T et al. netmeta: Network Meta-Analysis using Frequentist Methods [Internet]. 2022. Available from: https://CRAN.R-project.org/package=netmeta.

[9] Béliveau A, Boyne DJ, Slater J, Brenner D, Arora P (2019). BUGSnet: an R package to facilitate the conduct and reporting of bayesian network Meta-analyses. BMC Med Res Methodol.

[10] Hamza T, Schwarzer G, Salanti G. crossnma: Cross-Design & Cross-Format Network Meta-Analysis and Regression [Internet]. 2023 [cited 2023 Aug 6]. Available from: https://cran.r-project.org/web/packages/crossnma/index.html.

[11] Hamza T, Chalkou K, Pellegrini F, Kuhle J, Benkert P, Lorscheider J (2023). Synthesizing cross-design evidence and cross-format data using network meta-regression. Res Synthesis Methods.

[12] Dias S, Sutton AJ, Welton NJ, Ades AE (2013). Evidence synthesis for decision making 3: Heterogeneity—Subgroups, Meta-regression, Bias, and Bias-Adjustment. Med Decis Making.

[13] Gelman A, Carlin JB, Stern HS, Dunson DB, Vehtari A, Rubin DB. Bayesian Data Analysis [Internet]. 0 ed. Chapman and Hall/CRC; 2013 [cited 2022 Sep 16]. Available from: https://www.taylorfrancis.com/books/9781439898208.

[14] Peters JL, Sutton AJ, Jones DR, Abrams KR, Rushton L (2008). Contour-enhanced meta-analysis funnel plots help distinguish publication bias from other causes of asymmetry. J Clin Epidemiol.

[15] Egger M, Smith GD, Schneider M, Minder C (1997). Bias in meta-analysis detected by a simple, graphical test. BMJ.

[16] Harbord RM, Egger M, Sterne JAC (2006). A modified test for small-study effects in meta-analyses of controlled trials with binary endpoints. Stat Med.

[17] Peters JL (2006). Comparison of two methods to Detect Publication Bias in Meta-analysis. JAMA.

[18] Moreno SG, Sutton AJ, Ades A, Stanley TD, Abrams KR, Peters JL (2009). Assessment of regression-based methods to adjust for publication bias through a comprehensive simulation study. BMC Med Res Methodol.

[19] Moreno SG, Sutton AJ, Turner EH, Abrams KR, Cooper NJ, Palmer TM (2009). Novel methods to deal with publication biases: secondary analysis of antidepressant trials in the FDA trial registry database and related journal publications. BMJ.

[20] Moreno SG, Sutton AJ, Thompson JR, Ades AE, Abrams KR, Cooper NJ (2012). A generalized weighting regression-derived meta-analysis estimator robust to small-study effects and heterogeneity: a REGRESSION-DERIVED META-ANALYSIS MODEL ROBUST TO SMALL-STUDY EFFECTS. Statist Med.

[21] Copas JB, Shi JQ (2001). A sensitivity analysis for publication bias in systematic reviews. Stat Methods Med Res.

[22] Metelli S, Chaimani A. NMAstudio: a fully interactive web-application for producing and visualising network meta-analyses. In Bern, Switzerland; 2021. Available from: https://www.nmastudioapp.com/.

[23] Owen RK, Bradbury N, Xin Y, Cooper N, Sutton A, MetaInsight (2019). An interactive web-based tool for analyzing, interrogating, and visualizing network meta-analyses using R-shiny and netmeta. Res Synthesis Methods.

