# Supplementary figures and images for: Semi-automated assessment of the risk of bias due to missing evidence in network meta-analysis: a guidance paper for the ROB-MEN web-application
Source: BMC Med Res Methodol. 2023 Oct 7;23:223. doi: 10.1186/s12874-023-02038-9 (PMC10559514; doi:10.1186/s12874-023-02038-9)

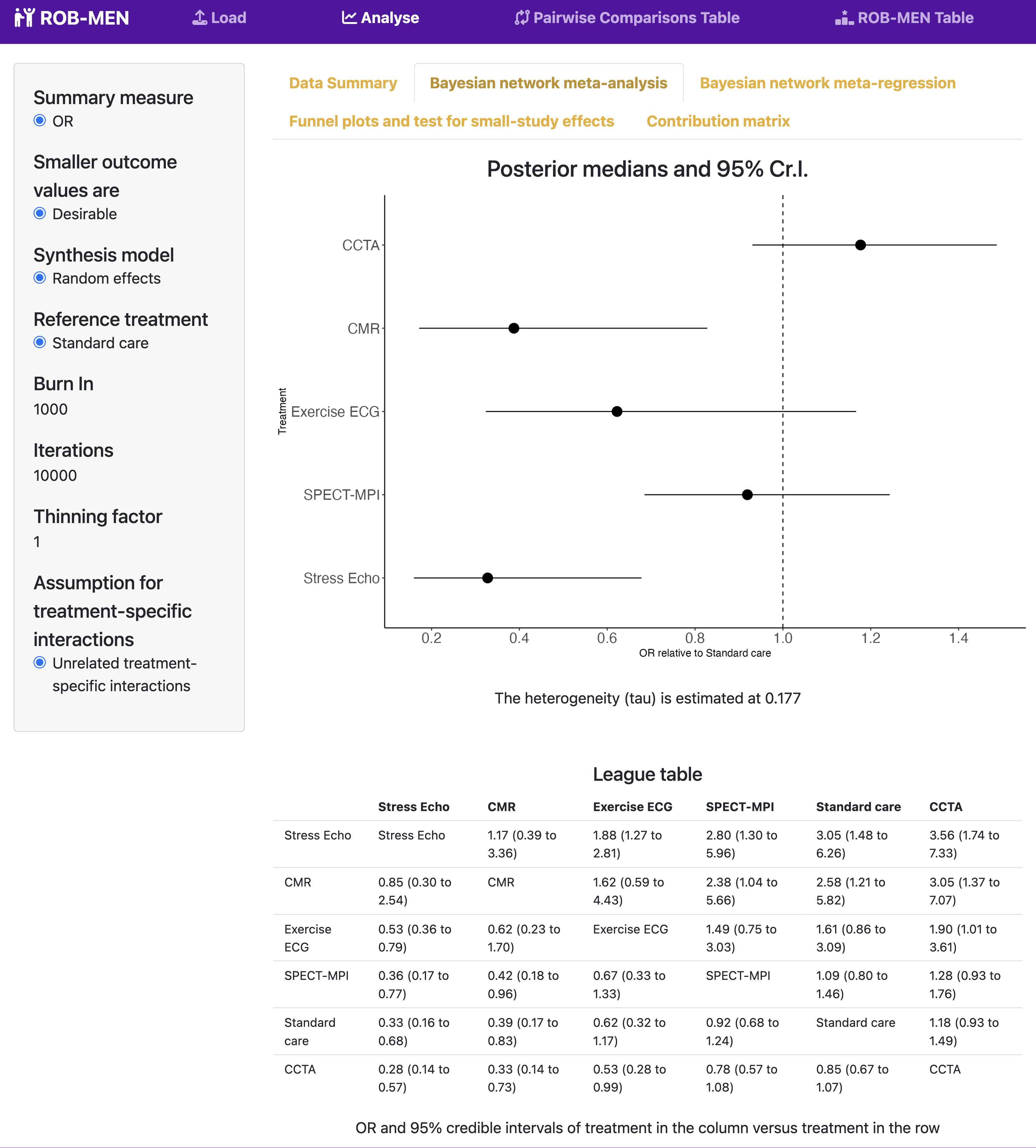

Supplement: Supplementary file 1 — Supplementary Material 1 [file 12874_2023_2038_MOESM1_ESM.png]

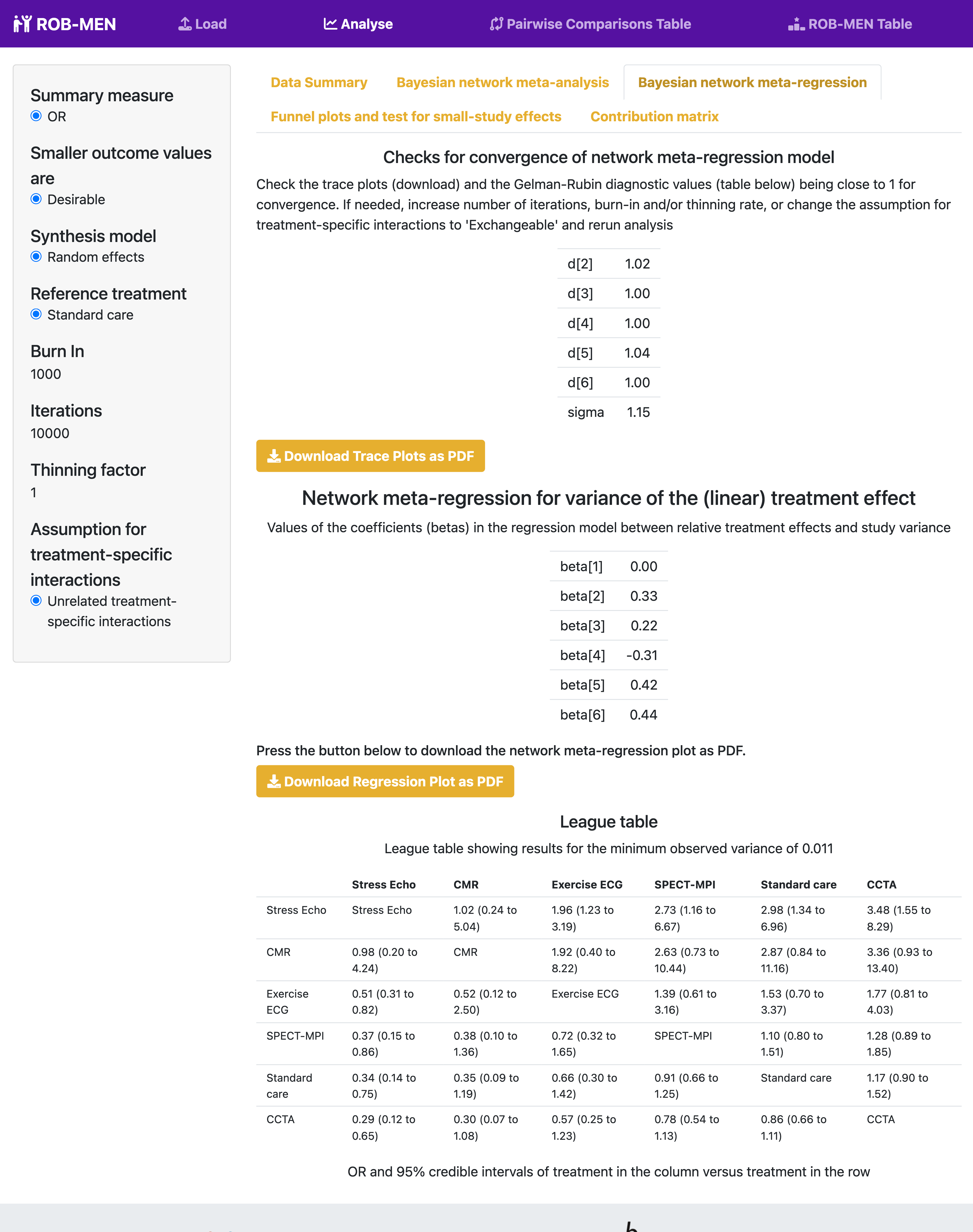

Supplement: Supplementary file 2 — Supplementary Material 2 [file 12874_2023_2038_MOESM2_ESM.png]
